# Supplementary figures and images for: The Distinctive Activation of Toll-Like Receptor 4 in Human Samples with Sepsis
Source: Cells. 2022 Sep 27;11(19):3020. doi: 10.3390/cells11193020 (PMC9563554; doi:10.3390/cells11193020)

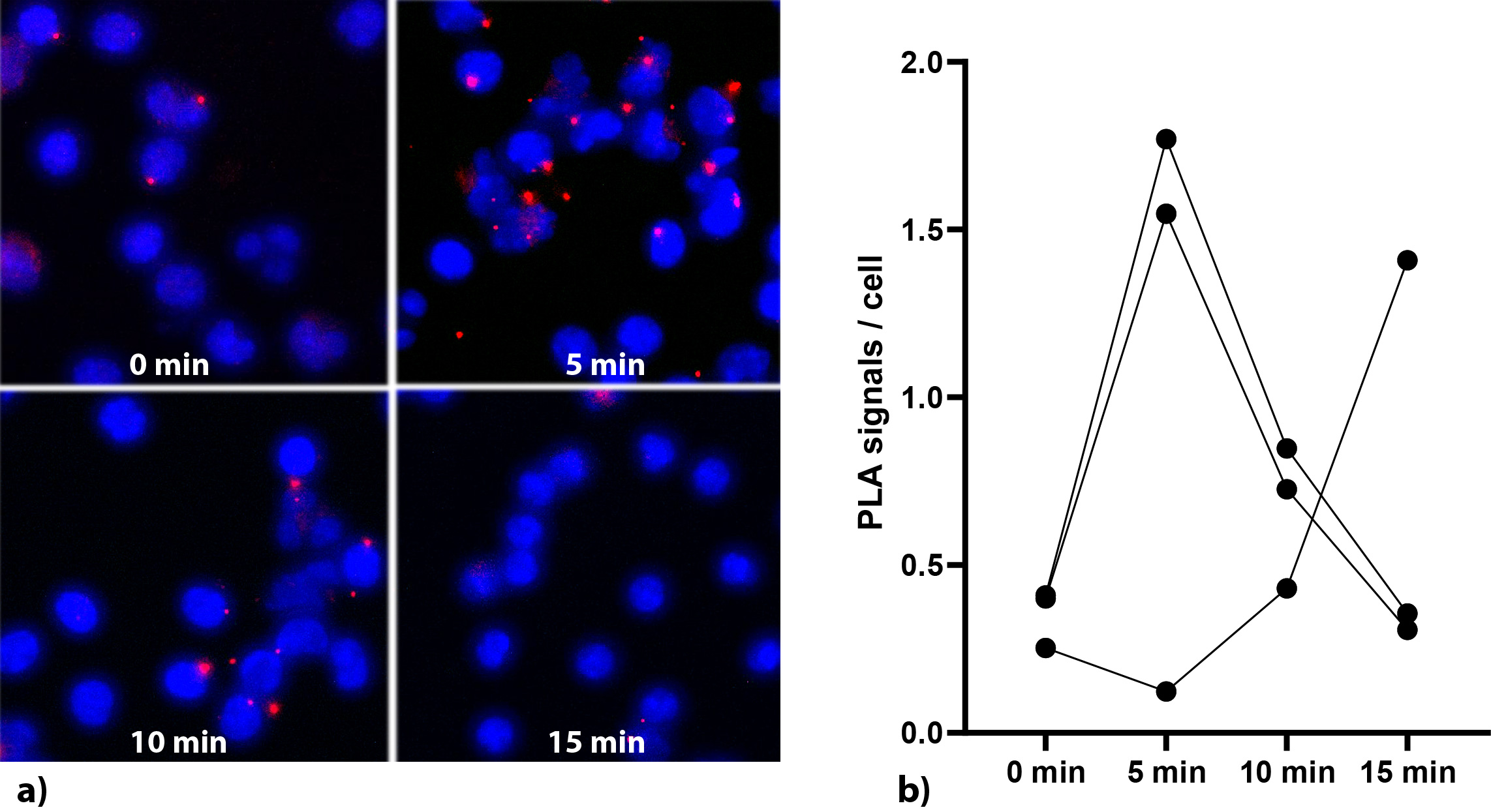

Supplement: Supplementary file 1 [file cells-11-03020-s001.zip › cells-1913994-Figure S1.jpg]
